# Exosomal circCOL1A1 promotes angiogenesis via recruiting EIF4A3 protein and activating Smad2/3 pathway in colorectal cancer

**DOI:** 10.1186/s10020-023-00747-x

**Published:** 2023-11-08

**Authors:** Gui Hu, Changwei Lin, Kai Gao, Miao Chen, Fei Long, Buning Tian

**Affiliations:** Department of Gastrointestinal Surgery, the Third Xiangya Hospital of Central South University, No. 138, Tongzipo Road, Changsha, 410013 Hunan Province P.R. China

**Keywords:** CircCOL1A1, Exosomes, EIF4A3, Smad2/3, Colorectal cancer, Angiogenesis

## Abstract

**Background:**

Colorectal cancer (CRC) is the third frequently diagnosed cancer with high incidence and mortality rate worldwide. Our previous report has demonstrated that circCOL1A1 (hsa_circ_0044556) functions as an oncogene in CRC, and Gene Ontology (GO) analysis has also revealed the strong association between circCOL1A1 and angiogenesis. However, the mechanism of circCOL1A1 or exosomal circCOL1A1 in CRC angiogenesis remains elusive.

**Methods:**

Purified exosomes from CRC cells were characterized by nanoparticle tracking analyzing, electron microscopy and western blot. qRT-PCR, immunohistochemistry or western blot were employed to test the expression of circCOL1A1, EIF4A3, Smad pathway and angiogenic markers. Cell proliferation of HUVECs was monitored by CCK-8 assay. The migratory and angiogenic capabilities of HUVECs were detected by wound healing and tube formation assay, respectively. Bioinformatics analysis, RNA immunoprecipitation (RIP), RNA pull-down and FISH assays were used to detect the interactions among circCOL1A1, EIF4A3 and Smad2/3 mRNA. The in vitro findings were verified in xenograft model.

**Results:**

CRC cell-derived exosomal circCOL1A1 promoted angiogenesis of HUVECs via recruiting EIF4A3. EIF4A3 was elevated in CRC tissues, and it stimulated angiogenesis of HUVECs through directly binding and stabilizing Smad2/3 mRNA. Moreover, exosomal circCOL1A1 promoted angiogenesis via inducing Smad2/3 signaling pathway in vitro, and it also accelerated tumor growth and angiogenesis in vivo.

**Conclusion:**

CRC cell-derived exosomal circCOL1A1 promoted angiogenesis via recruiting EIF4A3 and activating Smad2/3 signaling.

## Introduction

Colorectal cancer (CRC) remains the third common cancer globally with over 100,000 estimated new cases in the United States in 2021 (Siegel et al. 2021). It is also the second leading cause of cancer-related death worldwide (Siegel et al. 2020, 2021). It is well-established that angiogenesis is implicated in CRC development and progression, and the therapeutic agents targeted pro-angiogenic biomarker vascular endothelial growth factor (VEGF) have been incorporated into standard CRC treatment (Mousa et al. 2015). The targeted agents, such as anti-VEGF agent bevacizumab, enhance the efficacy of chemotherapy, leading to improved clinical outcomes in CRC (Douillard et al. 2000; Saltz et al. 2000). Unfortunately, the benefit of bevacizumab is limited for bevacizumab-resistant CRC patients (Mesange et al. 2014). Therefore, it is interesting to identify the novel biomarkers which can improve the therapeutic efficacy of the anti-angiogenic agents.

Circular RNAs (circRNAs) are a class of single-stranded RNAs that are lack of 5’-cap or 3’-poly (A) tail (Jeck and Sharpless et al. 2014). Emerging evidence indicates that circRNAs play crucial roles in a variety of human diseases, including CRC (Chen et al. 2016; Greene et al. 2017; Li et al. 2021). They exert either oncogenic or tumor suppressive roles by being microRNA (miRNA) sponges, transcription regulators, interacting with proteins and/or allowing for translation (Du et al. 2017; Hansen et al. 2013; Li et al. 2015; Pamudurti et al. 2017). A recent study has reported that circCOL1A1, also known as hsa_circ_0044556, is increased in gastric cancer (GC), and promotes GC growth and metastasis via miR-145/RABL3 axis (Ma et al. 2021). More importantly, our previous findings have illustrated that circCOL1A1 is highly expressed in CRC tissues and promotes CRC progression, and Gene Ontology (GO) analysis reveals that circCOL1A1 correlates with angiogenesis (Jing et al. 2020). Furthermore, cancer cell-derived exosomes stimulate angiogenesis in endothelial cells (Olejarz et al. 2020). However, the biological function and the underlying mechanism by which circCOL1A1 or exosomal circCOL1A1 exerts the pro-angiogenic role in CRC remain elusive.

Bioinformatics analysis (Circular RNA Interactome) predicted the direct interaction between circCOL1A1 and eukaryotic translation initiation factor 4A3 (EIF4A3). Interestingly, The Cancer Genome Atlas (TCGA) data from UALCAN platform showed that EIF4A3 was highly expressed in CRC. Previous researches have demonstrated that EIF4A3 is recruited by long non-coding RNAs (lncRNAs) or circRNAs in various cancers (Han et al. 2016; Xu et al. 2020; Yang et al. 2020; Ye et al. 2021; Zhu et al. 2021). For instance, lncRNA H19 promotes CRC growth through binding to EIF4A3 (Han et al. 2016). Circ_cse1l suppresses CRC proliferation via recruiting EIF4A3 (Xu et al. 2020). LINC00667 promotes non-small cell lung cancer (NSCLC) angiogenesis through EIF4A3-mediated stabilization of VEGFA (Yang et al. 2020). Moreover, bioinformatic analysis (Starbase) predicted Smad family members, namely Smad1, 2, 3, 4, 6, 7 and 9, as putative EIF4A3 binding partners. A number of studies have reported the pivotal roles of Smad pathway in angiogenesis (Hamik et al. 2006; Nakagawa et al. 2004), raising the possibility that circCOL1A1 might promote CRC angiogenesis via recruiting EIF4A3, possibly by activating Smad pathway.

In this study, we speculated that CRC cell-derived exosomal circCOL1A1 facilitated angiogenesis of human umbilical vein endothelial cells (HUVECs). CircCOL1A1 overexpression or exosomal circCOL1A1 promoted angiogenesis of HUVECs via recruiting EIF4A3. EIF4A3 was elevated in CRC tissues, and it promoted angiogenesis of HUVECs through binding and stabilizing Smad2/3 mRNA. In addition, exosomal circCOL1A1 promoted angiogenesis via activating Smad2/3 pathway in vitro, and it also accelerated tumor growth and angiogenesis in vivo. These data provide novel insights into anti-angiogenic targeted therapy for CRC.

## Materials and methods

### Clinical specimens

The CRC tissues and their normal counterparts were collected from 45 CRC patients at the Third Xiangya Hospital of Central South University. This study was approved by the Ethics Committee of the Third Xiangya Hospital of Central South University. Written consents were obtained from all participants. The clinicopathological characteristics of CRC patients recruited in the study were included in Table 1.

**Table 1:** Clinicopathological characteristics of patients with colorectal cancer

Characteristics	Cases
Age (years)	
< 60 years	20
≥ 60 years	25
Sex	
Male	18
Female	27
Tumor size (cm)	
<1.5	19
≥1.5	26
TNM stage	
I-II	14
III-IV	31
Histological type	
Adenocarcinoma	23
Mucinous adenocarcinoma	22
Liver metastasis	
Yes	29
No	16

### Cell culture and transfection

Human CRC cell lines (SW480 and HCT116) and HUVECs were obtained from the American Type Culture Collection (ATCC, Manassas, VA, USA). SW480 and HCT116 cells were cultured in RPMI1640 medium with 10% FBS (Gibco, Grand Island, NY, USA). All cells were maintained at 37 °C and 5% CO_2_. HUVECs were cultured in conditioned medium (CM) derived from CRC cells and HUVECs medium at a ratio of 1:2. The full-length of circCOL1A1 transcript was cloned into pLO5-ciR vector (Geneseed Biotech, Guangzhou, China). The lentiviral pLKO.1 vector containing short hairpin (shRNA) sequences, including sh-NC (negative control), sh-circCOL1A1 and sh-EIF4A3, were all obtained from Addgene (Watertown, MA, USA). Lentivirus production was conducted using Xfect transfection reagent (Takara, Dalian, China). The full-length of EIF4A3, Smad2 and Smad3 were cloned into pcDNA3.1 vector (Invitrogen). Transfection was performed using Lipofectamine 3000 (Invitrogen).

### Bioinformatics analyses

The binding between circCOL1A1 and EIF4A3 protein was predicted by Circular RNA Interactome (https://circinteractome.nia.nih.gov/). The expression of EIF4A3 in CRC and its association with CRC tumor stage and nodal metastasis were analyzed from TCGA data based on UALCAN (https://ualcan.path.uab.edu/index.html). The correlation between Smad2/3 expression and CRC overall survival was analyzed using GEPIA (http://gepia.cancer-pku.cn/), and the binding between EIF4A3 protein and Smad2/3 mRNA was predicted by Starbase (https://rnasysu.com/encori/).

### Exosome isolation and characterization

Exosomes were isolated from culture medium of CRC cells using ExoQuick-TC Exosome Precipitation Solution (System Biosciences, Palo Alto, CA, USA). In brief, the cell culture supernatant was collected after centrifugation, and incubated with ExoQuick-TC solution at 4 °C overnight. After centrifugation, exosome pellets were collected. For transmission electron microscopy (TEM) analysis, exosomes were placed on a carbon-coated copper grid and incubated for 3 min, followed by staining with 2% phosphotungstic acid. Exosomes were observed using Hitachi H-9500 TEM (Hitachi, Japan) as previously described (Pi et al. 2021). For nanopartical tracking analysis (NTA), exosomes were observed using Zetasizer (Malvern Panalytical, Malvern, UK).

### Exosome uptake assay

Exosome uptake assay was conducted using PKH26 Red Fluorescent Cell Linker Kit (Sigma-Aldrich, St Louis, MO, USA). Briefly, HUVECs (1 × 10^5^) were incubated with SW480 or HCT116 cell-derived exosomes (5 × 10^11^ particles/mL). After 24 h, HUVECs were harvested for immunofluorescence (IF) analysis. Images were acquired with a confocal microscope (Nikon, Tokyo, Japan).

### qRT-PCR

Total RNA was extracted using Trizol (Invitrogen). Reverse transcription was conducted using the High Capacity cDNA Reverse Transcription Kit (Thermo Fisher Scientific), and qRT-PCR was performed using SYBR Green PCR Master Mix (Thermo Fisher Scientific) on an ABI7500 system (ABI, Foster City, CA, USA). The expression of target gene was calculated using 2^–ΔΔCT^ method. GAPDH was used as an internal control. For RNA stability assay, HUVECs were treated with actinomycin D (5 µg/mL, Sigma-Aldrich) for 0, 3, 6, 9 and 12 h. The stabilities of Smad2/3 mRNA were analyzed by qRT-PCR. The primers were ordered from Sangon Biotech (Shanghai, China).

### Western blot

Proteins from exosomes, cells and tissues were prepared using RIPA lysis buffer (Beyotime, Shanghai, China). Proteins were separated by SDS-PAGE, and transferred onto nitrocellulose membranes (Millipore, Bedford, MA, USA). The blots were immunoblotted with primary antibodies, followed by the incubation with secondary antibody. Signals were visualized using ECL substrate (Sigma-Aldrich). Antibodies used in western blot including: CD63 (ab134045, Abcam), TSG101 (ab125011, Abcam), CD9 (ab236630, Abcam), Smad1 (ab126761, Abcam), Smad2 (ab33875, Abcam), Smad3 (ab208182, Abcam), Smad4 (ab40759, Abcam), Smad6 (ab273106, Abcam), Smad7 (ab216428, Abcam), p-Smad2 (ab188334, Abcam), p-Smad3 (ab63403, Abcam), VEGFR (ab11939, Abcam), EIF4A3 (ab32485, Abcam), and GAPDH (ab8245, Abcam).

### RNA immunoprecipitation (RIP) assay

RIP was performed using Magna RIP RNA-Binding Protein Immunoprecipitation Kit (Millipore) as described (Huang et al. 2020). Briefly, cells were lysed with RIP buffer. Anti-EIF4A3 antibody (ab32485, Abcam) or normal rabbit IgG-conjugated beads were incubated with cell lysates at 4 °C overnight. The enrichment of circCOL1A1 or Smad2/3 mRNA was detected by qRT-PCR.

### RNA pull-down assay

RNA pull-down assay was conducted using Pierce Magnetic RNA-protein pull-down Kit (Thermo Fisher Scientific). In brief, desthiobiotin-labelled circCOL1A1 was conjugated to streptavidin magnetic beads. The probe-conjugated beads were then incubated with cell lysates at 4 °C overnight. RNA-protein complexes were eluted and subjected to western blot.

### RNA fluorescence in situ hybridization (FISH) and immunofluorescence (IF)

The FITC-labeled circCOL1A1 or Smad2/3 mRNA probe was synthesized by RiboBio (Guangzhou, China). HUVECs were fixed and permeabilized. RNA FISH was performed using the Fluorescent in Situ Hybridization Kit (RiboBio). The slices were co-stained with anti-EIF4A3 antibody, followed by the incubation with Alexa Fluor 555-conjugated secondary antibody. The localization of circCOL1A1, Smad2/3 mRNA and EIF4A3 were visualized using a Nikon confocal microscope (Nikon). The probes used in FISH were as follows:

CircCOL1A, AAACTGGCCCCCCTGGCCCTGTTGGTGTTC;

Smad2 mRNA, cctaacagaacttccgcctctggatgacta;

Smad3 mRNA, gatggagaaaccagtgaccaccagatgaac.

### Cell counting Kit-8 (CCK-8) assay

HUVECs (1 × 10^3^/well) were seeded into 96-well plates 12 h prior to the treatment. Cell proliferation was assessed at 48 h post-treatment by CCK-8 assay (Solarbio, Beijing, China). In brief, 10 µL CCK-8 solution was added into each well and incubated at 37 °C for 1 h. A490 was measured using a microplate reader (Thermo Fisher Scientific).

### Wound healing assay

HUVECs were seeded into 6-well plates 12 h prior to the treatment. The cell monolayer was scratched using a pipette tip. The cells were rinsed with PBS to remove the detached cells. After 24 h, the scratches were photographed using a microscope (Nikon). Wound closure ratio was measured using the following formula: (W_0 h_‑W_24 h_)/W_0 h_×100%, where W is the width.

### Tube formation assay

Angiogenesis was assessed by tube formation assay as described (Qiu et al. 2021). In brief, HUVECs were seeded onto Matrigel (Corning, Corning, NY, USA)-coated plates. Tube-like structures were photographed 48 h post-treatment using a microscope (Nikon), and the number of tube branches was analyzed using ImageJ software (NIH).

### Animal study

All animal studies were approved by the Ethics Committee of the Third Xiangya Hospital of Central South University. Male BALB/c nude mice (6-week-old, n = 7 per group) were purchased from SLAC Laboratory Animal Center (Shanghai, China). SW480 or HCT116 cells (1 × 10^6^) were subcutaneously injected into the flank of mice. For each CRC cell line-derived xenograft model, mice were randomly divided into five groups: control, OE-NC-Exos, OE-circCOL1A1-Exos, sh-NC-Exos and sh-circCOL1A1-Exos. At day 2 post-inoculation, 1 × 10^12^ exosome particles (suspended in 100 µL PBS) were administered on mice via tail vein twice a week. Tumor size was monitored and determined as follows: volume = 1/2×length×width^2^. On day 28, xenograft tumors were dissected and weighted, followed by immunohistochemistry (IHC), qRT-PCR, and western blot analyses.

### IHC analysis

IHC was performed as previously described (Yuan et al. 2021). The paraffin-embedded sections were deparaffinized and rehydrated. After antigen retrieval, the sections were then incubated with primary antibodies, followed by the incubation with HRP-conjugated secondary antibody. The immunoreactivities were visualized using DAB substrate (Thermo Fisher Scientific). Antibodies used in IHC analysis were anti-Ki-67 (ab15580, Abcam), anti-E-cadherin (ab231303, Abcam), anti-N-cadherin (ab76011, Abcam), anti-CD31 (ab124432, Abcam) and anti-VEGFR (ab2349, Abcam).

### Statistical analysis

Data were presented as mean ± standard deviation (SD). All experiments were repeated independently at least 3 times. Statistical analysis was conducted using GraphPad Prism software 7.0 (GraphPad, La Jolla, CA, USA). One-way analysis of variance (ANOVA) followed by Tukey’s post hoc test or Student’s *t*-test was employed for multiple-group comparison or two-group comparison, respectively. *P* < 0.05 was considered statistically significant.

## Results

### CRC cell-derived exosomes promote angiogenesis of HUVECs

**Fig. 1:**
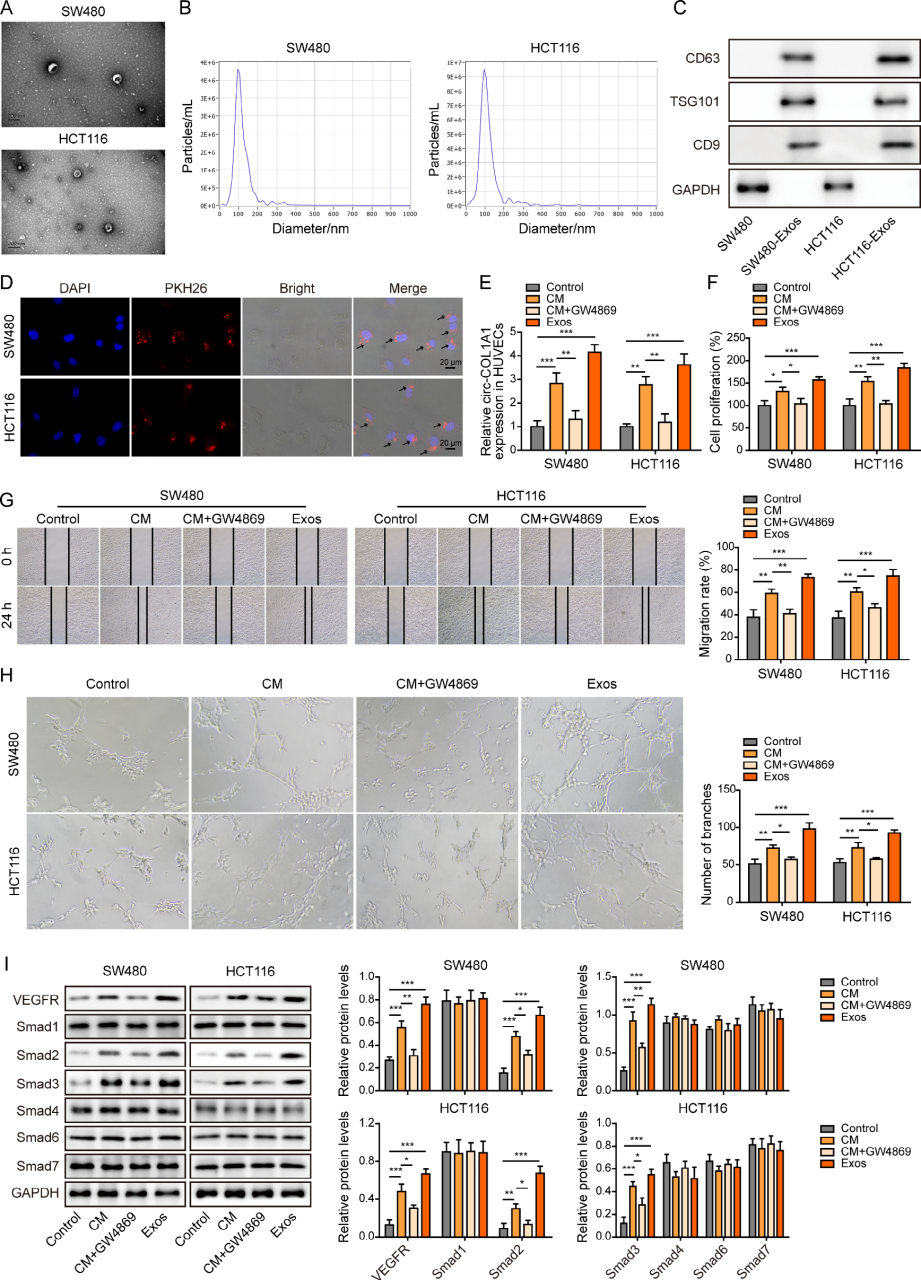
CRC cell-derived exosomes promote angiogenesis of HUVECs. (**A**) TEM analysis of SW480 or HCT116 cell-derived exosomes. (**B**) NTA of SW480 or HCT116 cell-derived exosomes. (**C**) Exosome markers CD63, TSG101 and CD9 were detected by western blot. (**D**) The uptake of PKH26-labeled exosomes was examined by confocal microscopy. Red: PKH26; Blue: DAPI. Scale bar, 20 μm. (**E**) The circCOL1A1 level in HUVECs was determined by qRT-PCR. (**F**) Cell proliferation was monitored by CCK-8 assay. (**G**) Cell migration was assessed by wound healing assay. (**H**) The in vitro angiogenesis was detected by tube formation assay. (**I**) The protein levels of VEGFR and Smad1/2/3/4/6/7 were detected by western blot. *, *P* < 0.05, **, *P* < 0.01, ***, *P* < 0.001

Our previous finding has illustrated that circCOL1A1 is highly expressed in SW480 and HCT116 cells (Jing et al. 2020). In order to study the role of CRC cell-derived exosomes, exosomes were isolated from the conditioned medium (CM) of SW480 and HCT116 cells. As shown in Fig. 1A-B, TEM and NTA showed small membrane vesicles with diameter ranging from 50 to 150 nm. Western blot revealed that SW480 or HCT116 cell-derived exosomes were positive for exosomal markers CD63, TSG101 and CD9, while SW480 or HCT116 cell lysates were negative for these markers (Fig. 1C). The cellular uptake of CRC cell-derived exosomes was observed by exosome uptake assay in which PKH26-labeled exosomes were found in HUVECs (Fig. 1D). We next examined the circCOL1A1 levels in HUVECs with different treatments. Compared with control group, circCOL1A1 was markedly increased in HUVECs treated with CM or exosomes (Exos) from CRC cells. The exosome inhibitor GW4869 (CM + GW4869) downregulated circCOL1A1 level, in comparison with CM group (Fig. 1E), suggesting that exosomes transported circCOL1A1 from CRC cells to HUVECs. In addition, functional experiments were then carried out to study the roles of CRC cell-derived exosomes. As presented in Fig. 1F-H, CRC cell-derived CM or Exos remarkably promoted the proliferative, migratory and tube forming capabilities of HUVECs, whereas GW4869 attenuated these effects as detected by functional assays. These findings suggest that the enhanced cell proliferation, metastasis and angiogenesis might be attributed to exosomal circCOL1A1 in CRC cell-derived CM. Western blot further showed that CRC cell-derived CM or Exos upregulated VEGFR, Smad2 and Smad3, but had no significant effect on Smad1, Smad4, Smad6 and Smad7 expression (Fig. 1I), indicating that VEGFR and Smad2/3 pathway might be involved in the enhanced HUVECs angiogenesis caused by CRC cell-derived exosomes. Collectively, these data suggest that CRC cell-derived exosomes promote angiogenesis of HUVECs.

### Exosomal circCOL1A1 promotes angiogenesis of HUVECs

**Fig. 2:**
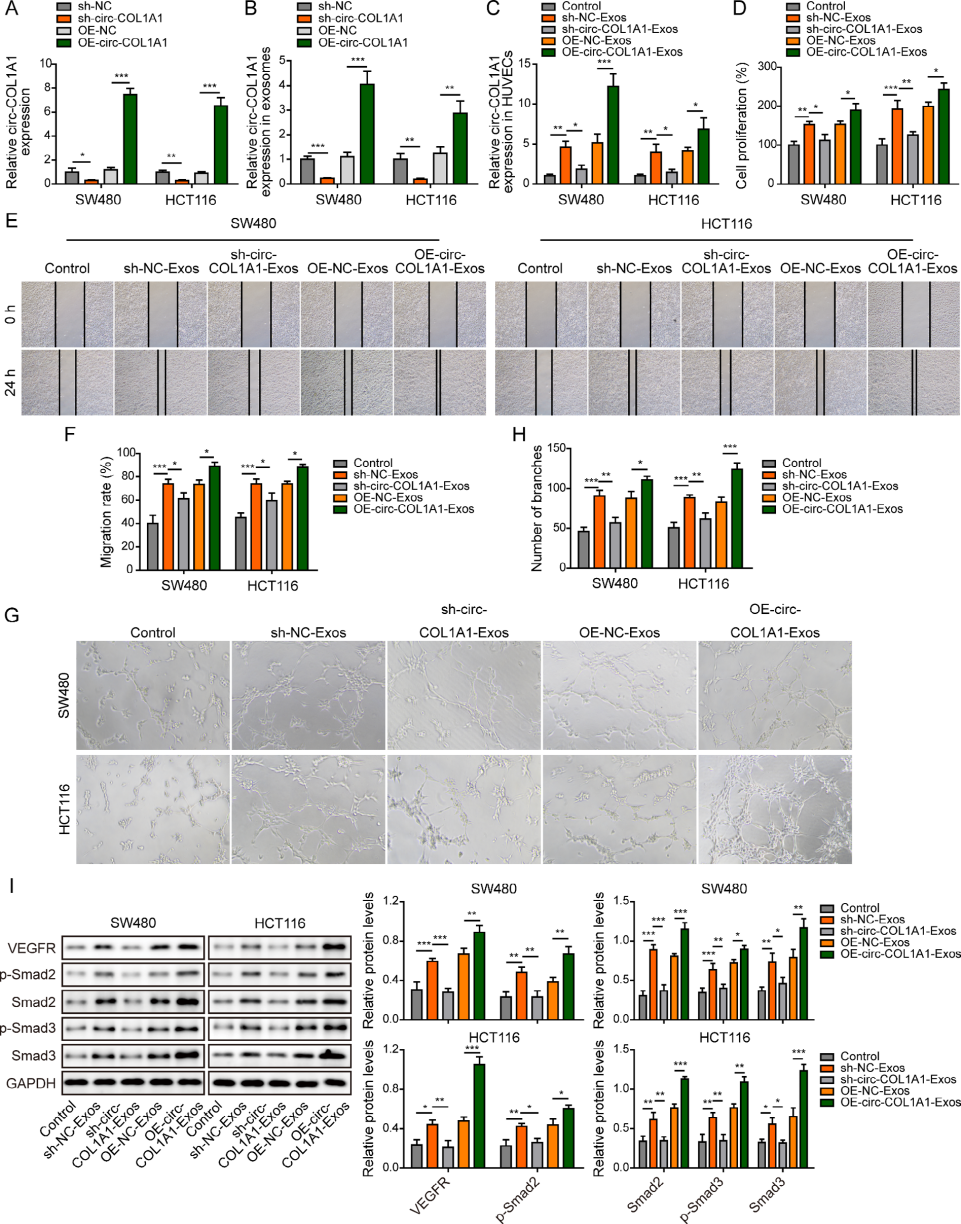
Exosomal circCOL1A1 promotes angiogenesis of HUVECs. SW480 and HCT116 cells were transfected with sh-NC, sh-circCOL1A1, OE-NC and OE-circCOL1A1. HUVECs were treated with the designated exosomes. The circCOL1A1 level in SW480 and HCT116 cells (**A**) or CRC cell-derived exosomes (**B**) was determined by qRT-PCR. (**C**) The circCOL1A1 level in HUVECs was determined by qRT-PCR. (**D**) Cell proliferation was monitored by CCK-8 assay. (**E-F**) Cell migration was assessed by wound healing assay. (**G-H**) The in vitro angiogenesis was detected by tube formation assay. (**I**) The protein levels of VEGFR and p-Smad2, Smad2, p-Smad3, and Smad3 were detected by western blot. *, *P* < 0.05, **, *P* < 0.01, ***, *P* < 0.001

We next thought to study whether exosomal circCOL1A1 played a pivotal role in HUVECs angiogenesis. Gain- and loss-of function studies were then respectively conducted. As expected, knockdown or overexpression of circCOL1A1 resulted in a remarkable reduction or induction of circCOL1A1 in both CRC cells and CRC cell-derived exosomes, respectively (Fig. 2A-B). HUVECs were then incubated with the designated exosomes, and circCOL1A1 level in HUVECs was not obviously upregulated after treated exosomes from circCOL1A1-knockdown group. By contrast, exosomes of overexpression of circCOL1A1 (OE-circCOL1A1-Exos) exerted an opposite effect (Fig. 2C). The treatment of OE-circCOL1A1-Exos greatly accelerated cell proliferation of HUVECs, whereas sh-circCOL1A1-Exos failed to enhance cell growth compared with corresponding controls (Fig. 2D). Wound healing (Fig. 2E-F) and tube formation (Fig. 2G-H) assays showed that OE-circCOL1A1-Exos promoted cell migratory and tube forming capacities of HUVECs, while sh-circCOL1A1-Exos failed to facilitate cell migration and in vitro angiogenesis (Fig. 2E-H). To further validate whether VEGFR or Smad2/3 signaling was implicated in exosomal circCOL1A1-mediated regulation of angiogenesis, western blot was performed. As shown in Fig. 2I, exosomes from knockdown or overexpression of circCOL1A1 group led to downregulation or upregulation of VEGFR, phosphorylated and total Smad2/3 compared to corresponding controls, respectively. Taken together, these findings indicate that exosomal circCOL1A1 promotes angiogenesis of HUVECs, possibly via Smad2/3 signaling.

### CircCOL1A1 directly binds to EIF4A3 in HUVECs

**Fig. 3:**
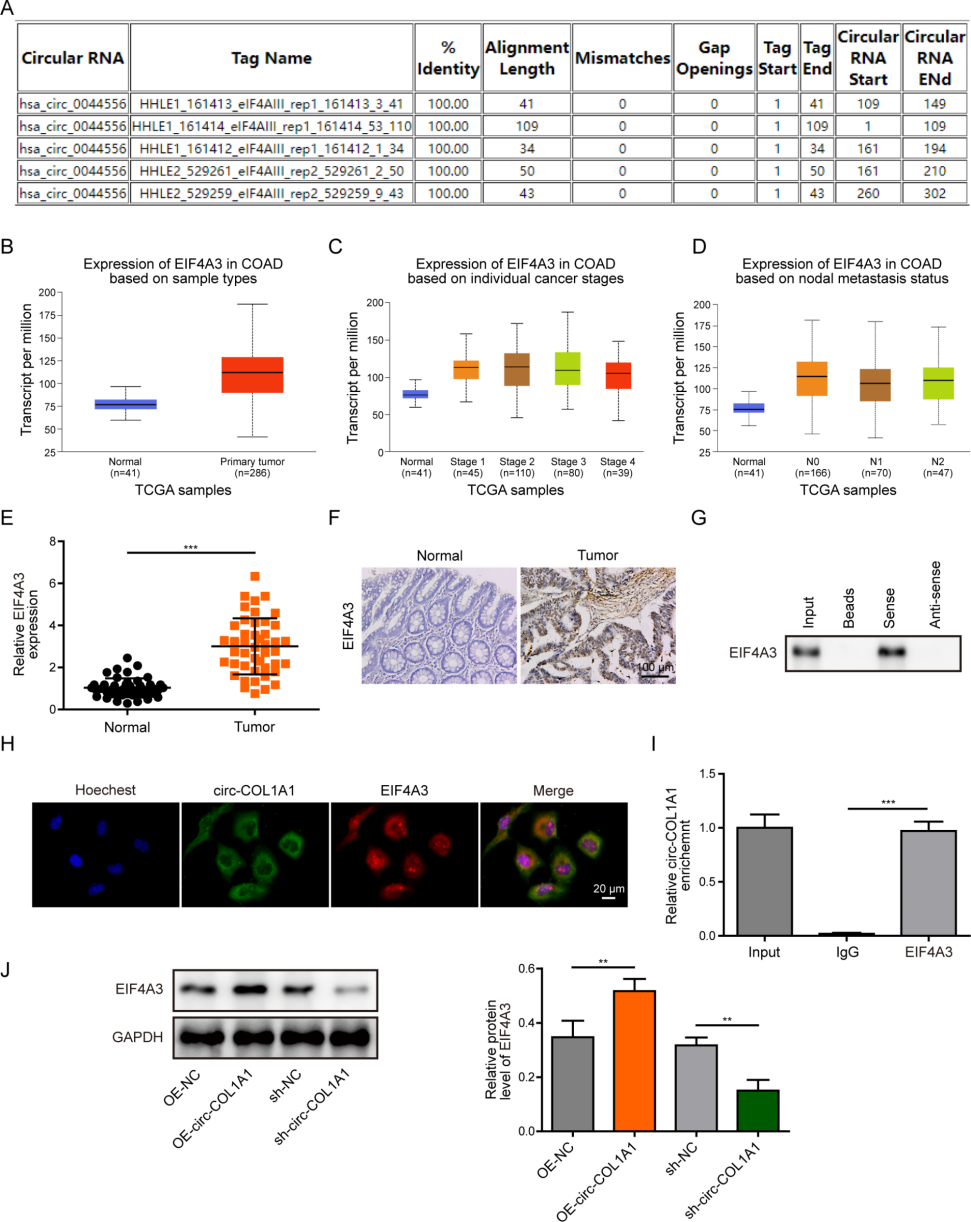
CircCOL1A1 directly binds to EIF4A3 protein in HUVECs. (**A**) Bioinformatics analysis of circCOL1A1-binding proteins by Circular RNA Interactome. (**B-D**) Data analyses on EIF4A3 expression based on TCGA data from UALCAN database. (**E**) The mRNA level of EIF4A3 in CRC tissues was determined by qRT-PCR. (**F**) The immunoreactivity of EIF4A3 in CRC tissues was detected by IHC. Scale bar, 100 μm. (**G**) The association between circCOL1A1 and EIF4A3 was detected by RNA pull-down assay. Anti-sense circCOL1A1 probe served as a negative control. (**H**) The co-localization of circCOL1A1 and EIF4A3 protein was assessed by RNA FISH and IF staining. Green, circCOL1A1; Red, EIF4A3; Blue, DAPI. Scale bar, 20 μm. (**I**) The association between circCOL1A1 and EIF4A3 was detected by RIP assay. Normal IgG served as a negative control. (**J**) The protein level of EIF4A3 in HUVECs was detected by western blot. *, *P* < 0.05, **, *P* < 0.01, ***, *P* < 0.001

Bioinformatics analysis (Circular RNA Interactome) revealed that circCOL1A1 bound to EIF4A3 protein directly (Fig. 3A). EIF4A3 was markedly increased in CRC tissues, and its level was positively associated with tumor stage and nodal metastasis based on TCGA data from UALCAN platform **(**Fig. 3B-D). Consistently, qRT-PCR and IHC staining unequivocally showed that EIF4A3 was elevated in CRC tissues compared with their normal counterparts (Fig. 3E-F). The direct interaction between circCOL1A1 and EIF4A3 was further validated by RNA pull-down, FISH/IF staining, and RIP. As presented in Fig. 3G, desthiobiotin-labelled circCOL1A1 pulled down EIF4A3 protein as detected by RNA pull-down assay. FISH/IF staining revealed the co-localization of circCOL1A1 and EIF4A3 protein in the cytoplasm (Fig. 3H). Conversely, antibody against EIF4A3 successfully enriched circCOL1A1 in HUVECs (Fig. 3I). Overexpression and knockdown experiments further showed that circCOL1A1 positively regulated EIF4A3 expression in HUVECs at protein level (Fig. 3J). Together, these data show that circCOL1A1 positively regulates EIF4A3 expression through direct binding to EIF4A3 in HUVECs.

### CircCOL1A1 promotes angiogenesis of HUVECs via recruiting EIF4A3

**Fig. 4:**
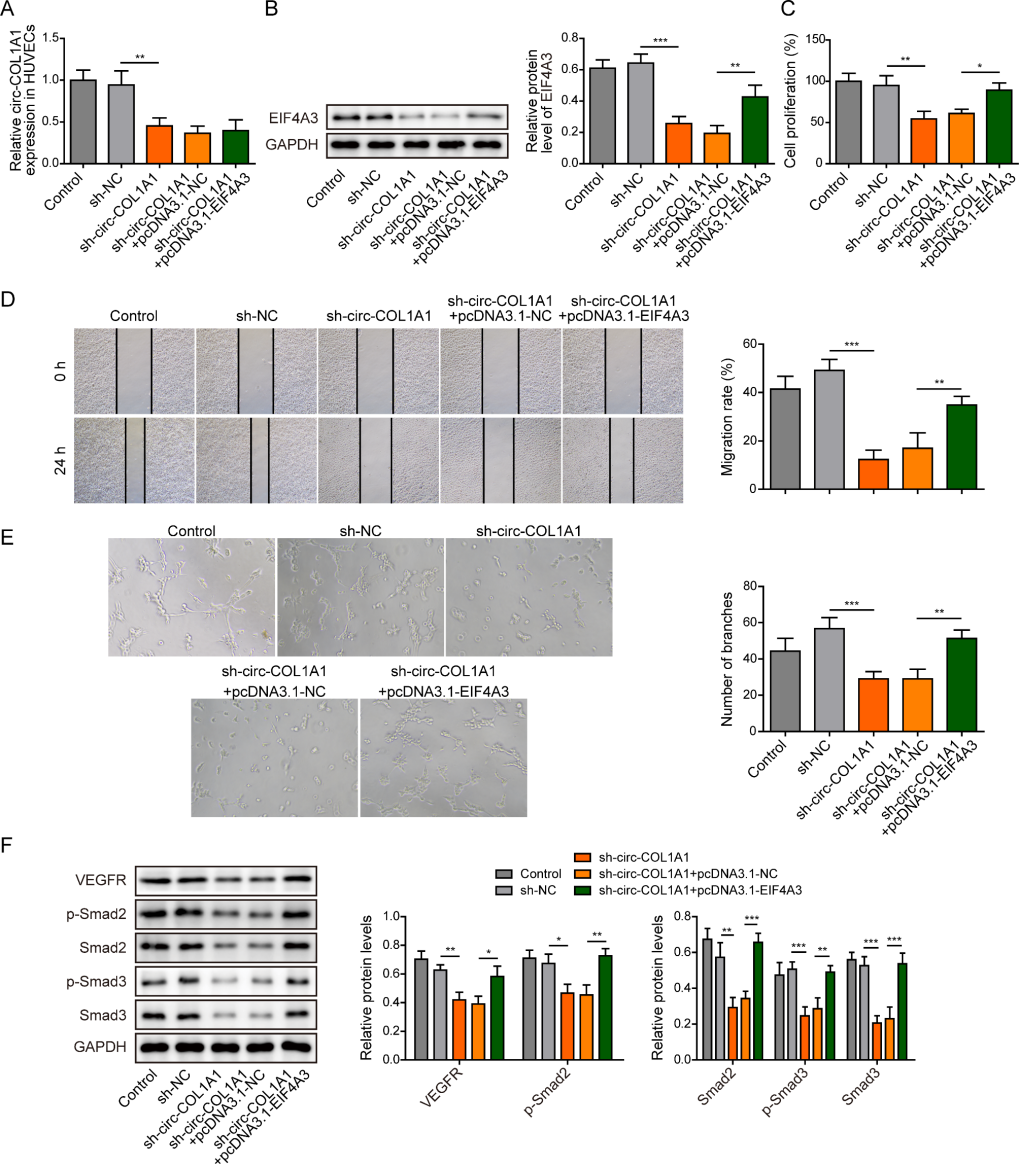
CircCOL1A1 promotes angiogenesis of HUVECs via recruiting EIF4A3. HUVECs were transfected with sh-NC, sh-circCOL1A1, sh-circCOL1A1 + pcDNA3.1-NC and sh-circCOL1A1 + pcDNA3.1-EIF4A3. (**A**) The circCOL1A1 level in HUVECs was determined by qRT-PCR. (**B**) The protein level of EIF4A3 in HUVECs was detected by western blot. (**C**) Cell proliferation was monitored by CCK-8 assay. (**D**) Cell migration was assessed by wound healing assay. (**E**) The in vitro angiogenesis was detected by tube formation assay. (**F**) The protein levels of VEGFR and p-Smad2, Smad2, p-Smad3, and Smad3 were detected by western blot. *, *P* < 0.05, **, *P* < 0.01, ***, *P* < 0.001

To further delineate the role of EIF4A3 in circCOL1A1-enhanced angiogenesis, rescued experiments were carried out. As presented in Fig. 4A, transfection of sh-circCOL1A1 led to remarkable decrease of circCOL1A1 in HUVECs, and co-transfection of sh-circCOL1A1 and pcDNA3.1-EIF4A3 caused no further change on circCOL1A1 level in comparison with transfection of sh-circCOL1A1 alone. As expected, the EIF4A3 protein level was decreased by circCOL1A1 knockdown, while EIF4A3 was significantly increased in sh-circCOL1A1 + pcDNA3.1-EIF4A3 group (Fig. 4B). Overexpression of EIF4A3 partially rescued sh-circCOL1A1-impaired cell proliferation and migration in HUVECs (Fig. 4C-D). Additionally, reduced capability to form capillary-like structure was observed in circCOL1A1-knockdown HUVECs, whereas EIF4A3 overexpression attenuated the suppressive effect on tube formation (Fig. 4E). Furthermore, overexpression of EIF4A3 reversed the effects of sh-circCOL1A1 on VEGFR, phosphorylated and total Smad2/3 in HUVECs (Fig. 4F). These findings suggest that circCOL1A1 promotes angiogenesis of HUVECs via recruiting EIF4A3.

### Exosomal circCOL1A1 promotes angiogenesis of HUVECs via recruiting EIF4A3

**Fig. 5:**
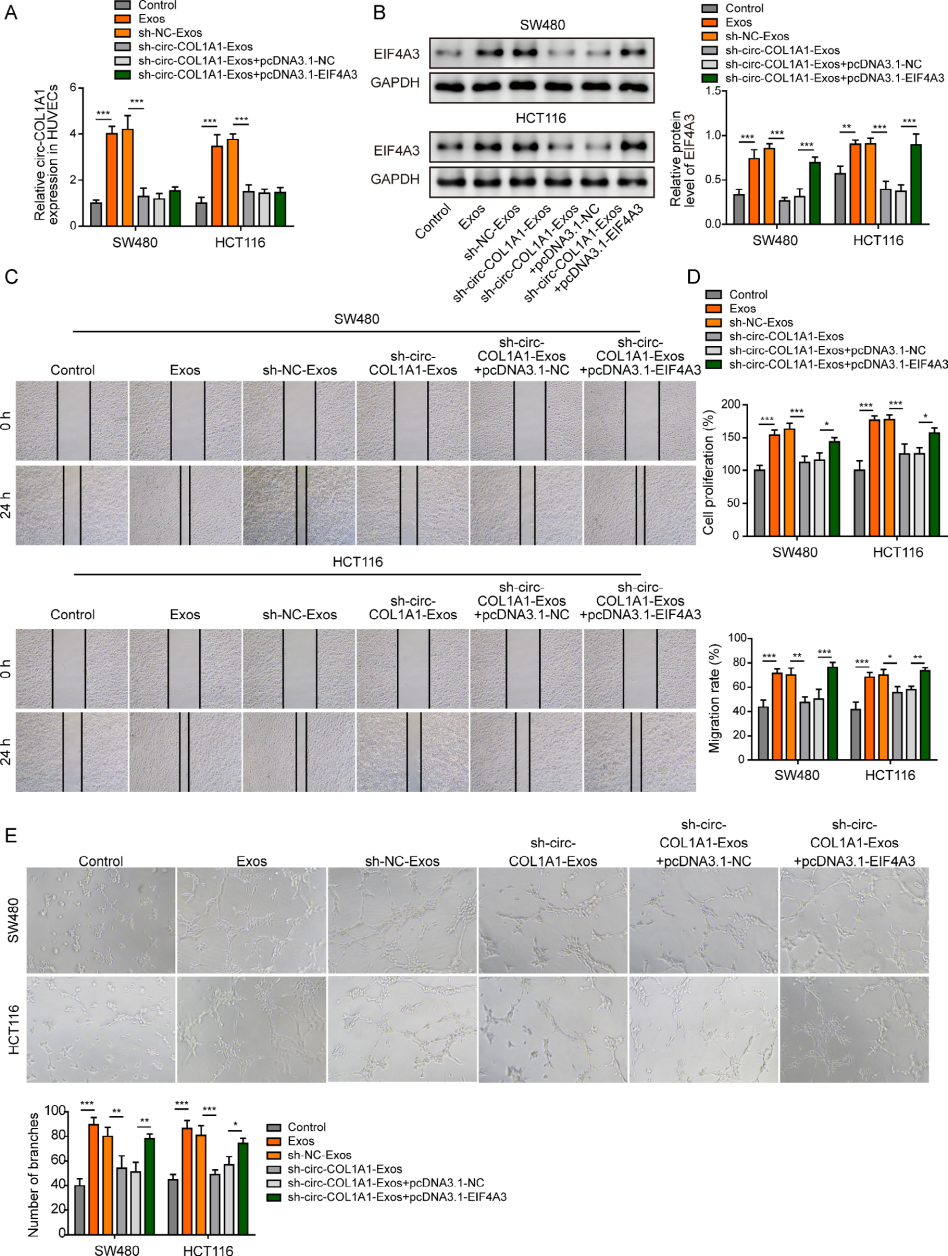
Exosomal circCOL1A1 promotes angiogenesis of HUVECs via recruiting EIF4A3. HUVECs were transfected with pcDNA3.1-NC or pcDNA3.1-EIF4A3, followed by treatment with CRC cell-derived exosomes. (**A**) The circCOL1A1 level in HUVECs was determined by qRT-PCR. (**B**) The protein level of EIF4A3 in HUVECs was detected by western blot. (**C**) Cell migration was assessed by wound healing assay. (**D**) Cell proliferation was monitored by CCK-8 assay. (**E**) The in vitro angiogenesis was detected by tube formation assay. *, *P* < 0.05, **, *P* < 0.01, ***, *P* < 0.001

**Fig. 6:**
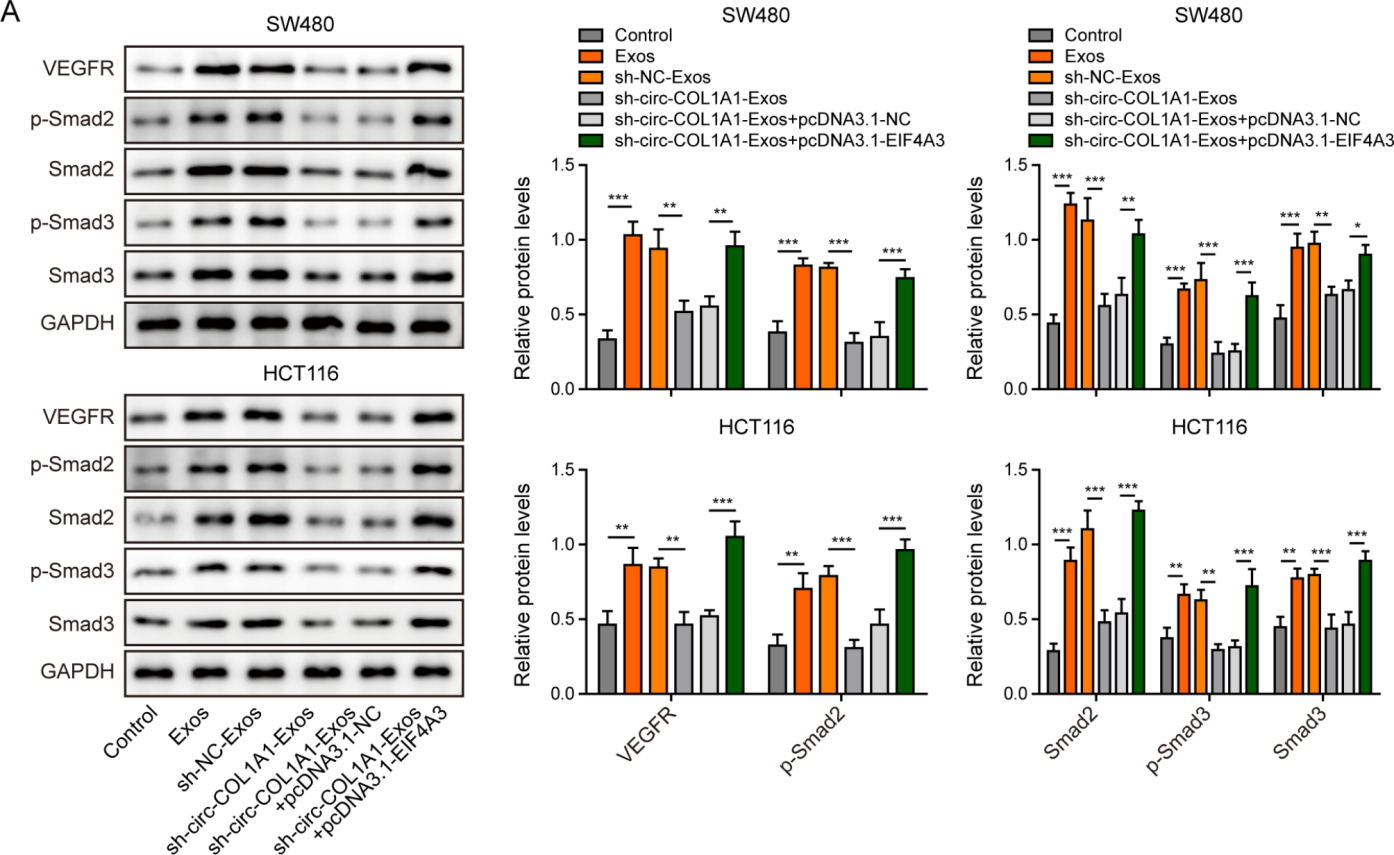
Exosomal circCOL1A1 promotes angiogenesis through Smad2/3 pathway. HUVECs were transfected with pcDNA3.1-NC or pcDNA3.1-EIF4A3, followed by treatment with CRC cell-derived exosomes. (**A**) The protein levels of VEGFR and p-Smad2, Smad2, p-Smad3, and Smad3 were detected by western blot. *, *P* < 0.05, **, *P* < 0.01, ***, *P* < 0.001

We next investigated whether EIF4A3 also acted as a key player in exosomal circCOL1A1-accelarated angiogenesis in HUVECs. HUVECs were transfected with pcDNA3.1-NC or pcDNA3.1-EIF4A3, followed by the treatment with CRC cell-derived exosomes. qRT-PCR showed that CRC cell-derived exosomes increased circCOL1A1 level in HUVECs, while silencing of circCOL1A1 abrogated this effect in the presence or absence of EIF4A3 overexpression (Fig. 5A). Exosomes from silencing of circCOL1A1 group decreased EIF4A3 expression, while EIF4A3 overexpression resulted in a markedly induction of EIF4A3 at protein level **(**Fig. 5B). Similarly, the cell proliferation, migration and tube formation were attenuated by sh-circCOL1A1-Exos in HUVECs, while EIF4A3 overexpression partially rescued the suppressive effects (Fig. 5C-E). Moreover, the upregulation of VEGFR, phosphorylated and total Smad2/3 mediated by CRC cell-derived exosomes were abolished by sh-circCOL1A1, whereas EIF4A3 overexpression reversed these negative effects on VEGFR and Smad2/3 expression (Fig. 6A). Collectively, these data indicate that exosomal circCOL1A1 also promotes angiogenesis of HUVECs via recruiting EIF4A3.

### EIF4A3 binds to Smad2/3 mRNA directly

**Fig. 7:**
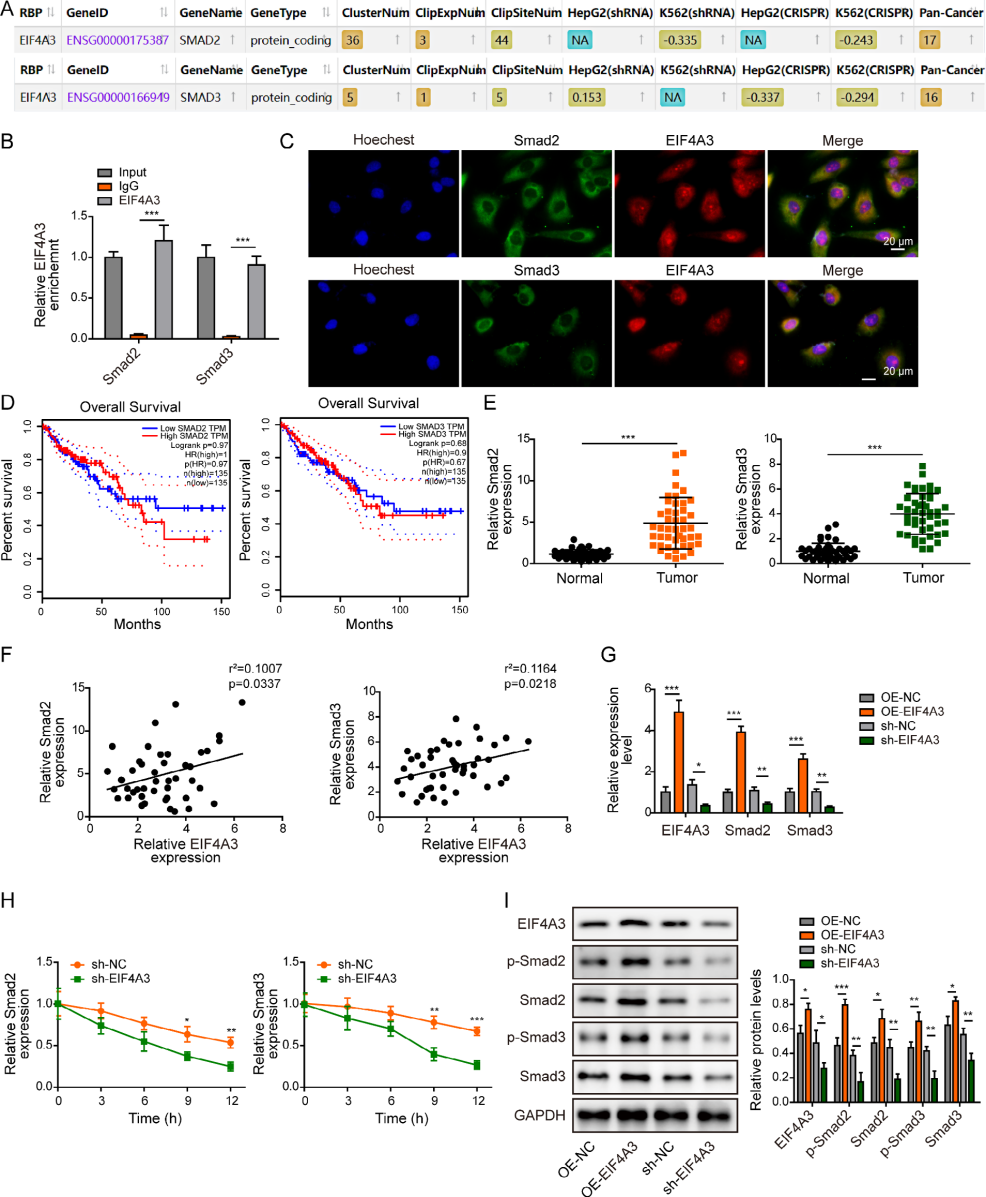
EIF4A3 binds Smad2/3 mRNA directly. (**A**) The putative binding sites between EIF4A3 protein and Smad2/3 mRNA were predicted by bioinformatics analysis (Starbase). (**B**) The direct interaction between EIF4A3 protein and Smad2/3 mRNA was assessed by RIP assay. Normal IgG served as a negative control. (**C**) The co-localization of Smad2/3 mRNA and EIF4A3 protein was assessed by RNA FISH and IF staining. Green, Smad2/3 mRNA; Red, EIF4A3; Blue, DAPI. Scale bar, 20 μm. (**D**) Correlation of Smad2/3 expression with overall survival of CRC patients was from GEPIA database. (**E**) The mRNA levels of Smad2 and Smad3 in CRC tissues were determined by qRT-PCR. (**F**) Pearson correlation analysis between EIF4A3 and Smad2, as well as between EIF4A3 and Smad3 in CRC tissues. (**G**) The mRNA levels of EIF4A3 and Smad2/3 in HUVECs were detected by qRT-PCR. (**H**) The stabilities of Smad2/3 mRNA were assessed by RNA stability assay. (**I**) The protein levels of EIF4A3 and p-Smad2, Smad2, p-Smad3, and Smad3 were detected by western blot. *, *P* < 0.05, **, *P* < 0.01, ***, *P* < 0.001

Bioinformatics analysis (Starbase) predicted that EIF4A3 directly bound to Smad2/3 mRNA (Fig. 7A). The direct association between EIF4A3 and Smad2/3 mRNA was validated by RIP assay. As shown in Fig. 7B, anti-EIF4A3 antibody successfully enriched Smad2 or Smad3 mRNA in HUVECs. The co-localization of EIF4A3 protein and Smad2/3 mRNA was further confirmed by FISH/IF staining (Fig. 7C). It also revealed that CRC patients with high Smad2/3 expression were associated with unfavorable overall survival, although no significant difference was observed (Fig. 7D). qRT-PCR showed that Smad2 and Smad3 were elevated in CRC tissues compared with matched normal counterparts (Fig. 7E). Pearson correlation analysis illustrated that there was a positive correlation between EIF4A3 and Smad2, as well as between EIF4A3 and Smad3 in CRC tissues (Fig. 7F). Transfection of pcDNA3.1-EIF4A3 or sh-EIF4A3 successfully increased or decreased EIF4A3 mRNA and protein levels in HUVECs, respectively (Fig. 7G and **I**). qRT-PCR results showed that overexpression of EIF4A3 increased Smad2/3 mRNA levels, while silencing of EIF4A3 led to the reduction of Smad2/3 mRNA **(**Fig. 7G). RNA stability assay revealed that knockdown of EIF4A3 impaired the stabilities of Smad2/3 mRNA in the presence of actinomycin D (Fig. 7H). Overexpression of EIF4A3 increased phosphorylated and total Smad2/3 levels, while lack of EIF4A3 exerted an opposite effect in HUVECs (Fig. 7I). These data suggest that EIF4A3 is elevated in CRC tissues, and it directly binds to Smad2/3 mRNA to promote their stabilities in HUVECs.

### EIF4A3 promotes angiogenesis of HUVECs through binding to Smad2/3 mRNA

**Fig. 8:**
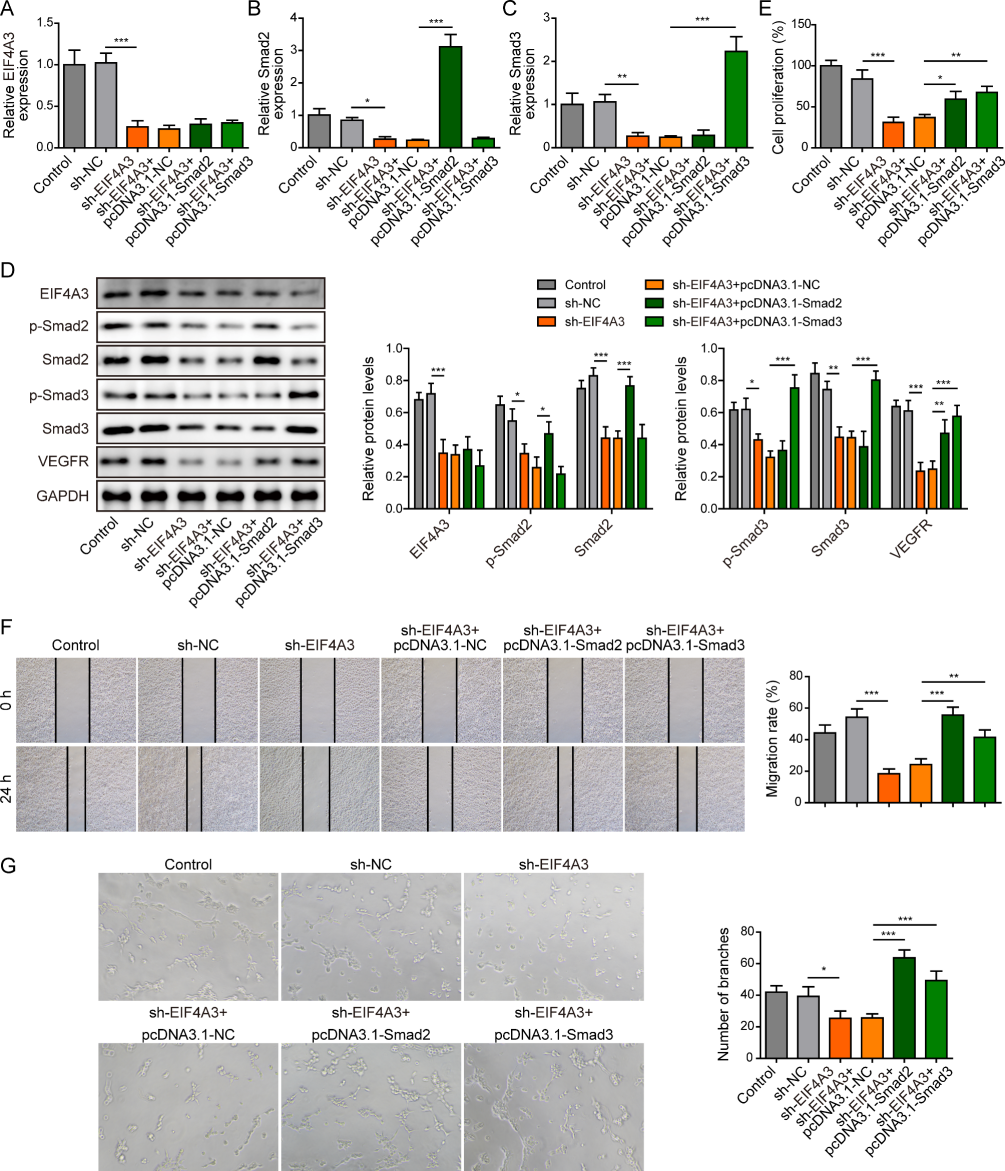
EIF4A3 promotes angiogenesis of HUVECs through binding to Smad2/3 mRNA. HUVECs were transfected with sh-NC, sh-EIF4A3, sh-EIF4A3 + pcDNA3.1-NC, sh- EIF4A3 + pcDNA3.1-Smad2 and sh-EIF4A3 + pcDNA3.1-Smad3. (**A**) The mRNA level of EIF4A3 in HUVECs was detected by qRT-PCR. The mRNA levels of Smad2 (**B**) and Smad3 (**C**) were detected by qRT-PCR. (**D**) The protein levels of EIF4A3, p-Smad2, Smad2, p-Smad3, Smad3, and VEGFR were detected by western blot. (**E**) Cell proliferation was monitored by CCK-8 assay. (**F**) Cell migration was assessed by wound healing assay. (**G**) The in vitro angiogenesis was detected by tube formation assay. *, *P* < 0.05, **, *P* < 0.01, ***, *P* < 0.001

To investigate the mechanism underlying EIF4A3-regulated HUVECs angiogenesis, gain- and loss-of function experiments were respectively performed. As shown in Fig. 8A and **D**, loss of EIF4A3 led to a marked reduction of EIF4A3, and overexpression of Smad2/3 caused no further change on EIF4A3 level in HUVECs. As expected, silencing of EIF4A3 suppressed the expression of phosphorylated and total Smad2/3, while Smad2 or Smad3 overexpression increased their expression levels, respectively (Fig. 8B-D). Moreover, overexpression of Smad2/3 reversed sh-EIF4A3-downregulated VEGFR in HUVECs (Fig. 8D). Functional studies further illustrated that Smad2/3 overexpression abrogated sh-EIF4A3-inhibited cell proliferation (Fig. 8E**)**, migration (Fig. 8F) and tube formation (Fig. 8G) in HUVECs. Collectively, these data suggest that EIF4A3 promotes angiogenesis of HUVECs through binding to Smad2/3 mRNA.

### Exosomal circCOL1A1 promotes angiogenesis of HUVECs via activating Smad2/3 pathway

**Fig. 9:**
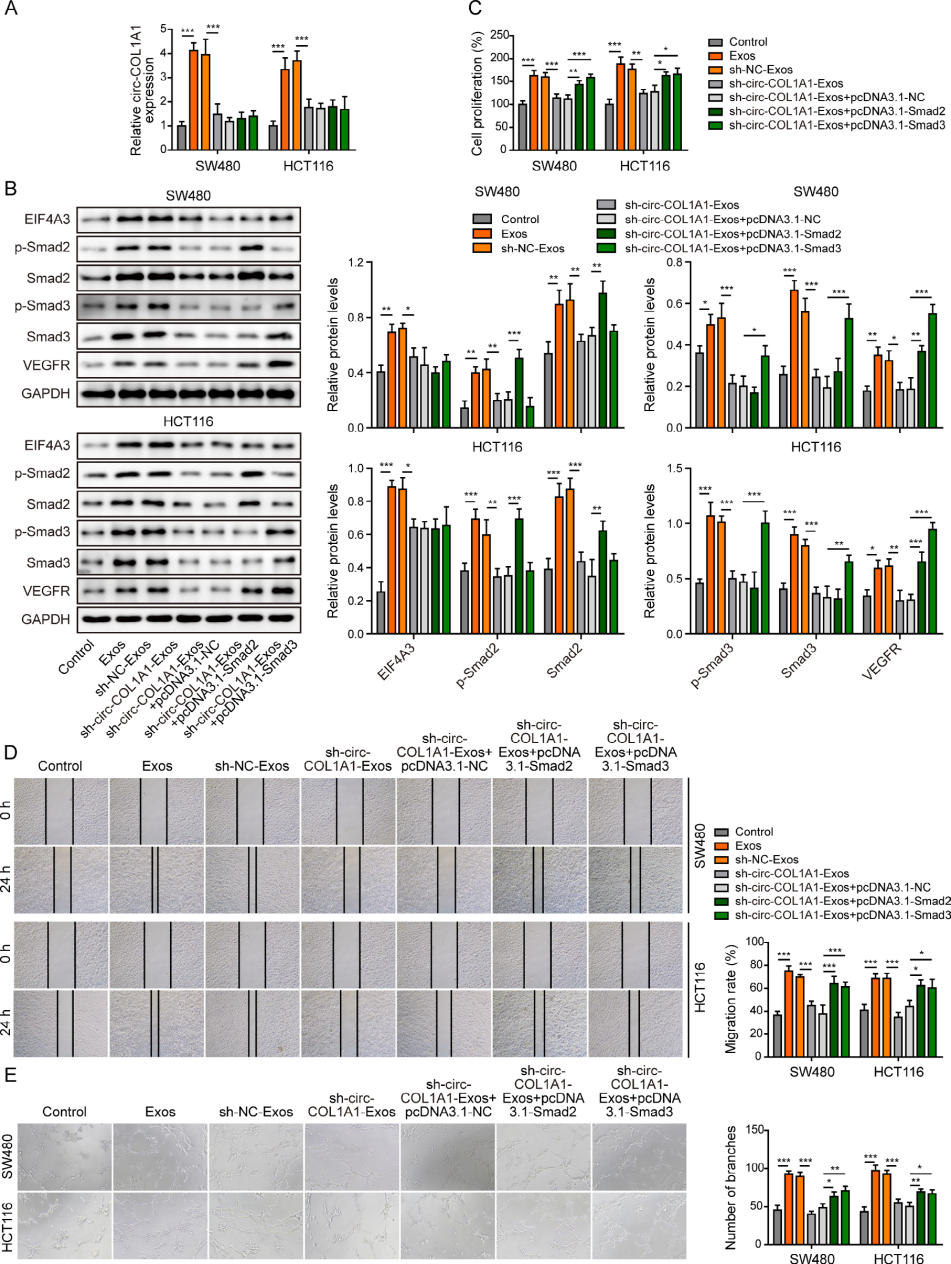
Exosomal circCOL1A1 promotes angiogenesis of HUVECs via activating Smad2/3 pathway. HUVECs were transfected with pcDNA3.1-NC, pcDNA3.1-Smad2 or pcDNA3.1-Smad3, followed by treatment with CRC cell-derived exosomes. (**A**) The circCOL1A1 level in HUVECs was determined by qRT-PCR. (**B**) The protein levels of EIF4A3, p-Smad2, Smad2, p-Smad3, Smad3, and VEGFR in HUVECs were detected by western blot. (**C**) Cell proliferation was monitored by CCK-8 assay. (**D**) Cell migration was assessed by wound healing assay. (**E**) The in vitro angiogenesis was detected by tube formation assay. *, *P* < 0.05, **, *P* < 0.01, ***, *P* < 0.001

To further test if Smad2/3 signaling was involved in exosomal circCOL1A1-enhanced HUVECs angiogenesis, HUVECs were transfected with Smad2 or Smad3 overexpression construct and treated with the designated exosomes. We next examined the circCOL1A1 and EIF4A3 expression levels in HUVECs. Consistently, sh-circCOL1A1 abolished exosomes-mediated induction of circCOL1A1 and EIF4A3, and co-transfection of Smad2/3 had no remarkable effect on circCOL1A1 and EIF4A3 levels (Fig. 9A-B). Western blot further confirmed the upregulation of phosphorylated and total Smad2/3 in Smad2/3-overexpressing HUVECs (Fig. 9B). In addition, sh-circCOL1A1 attenuated Exos- or sh-NC-Exos-upregulated VEGFR in HUVECs, and this effect was reversed by Smad2/3 overexpression (Fig. 9B). As expected, sh-circCOL1A1-Exos failed to promote cell proliferation, migration and tube formation, and these failures were rescued by Smad2/3 overexpression (Fig. 9C-E). These findings indicate that exosomal circCOL1A1 promotes angiogenesis of HUVECs via activating Smad2/3 pathway.

### Exosomal circCOL1A1 facilitates tumor growth and angiogenesis in vivo

**Fig. 10:**
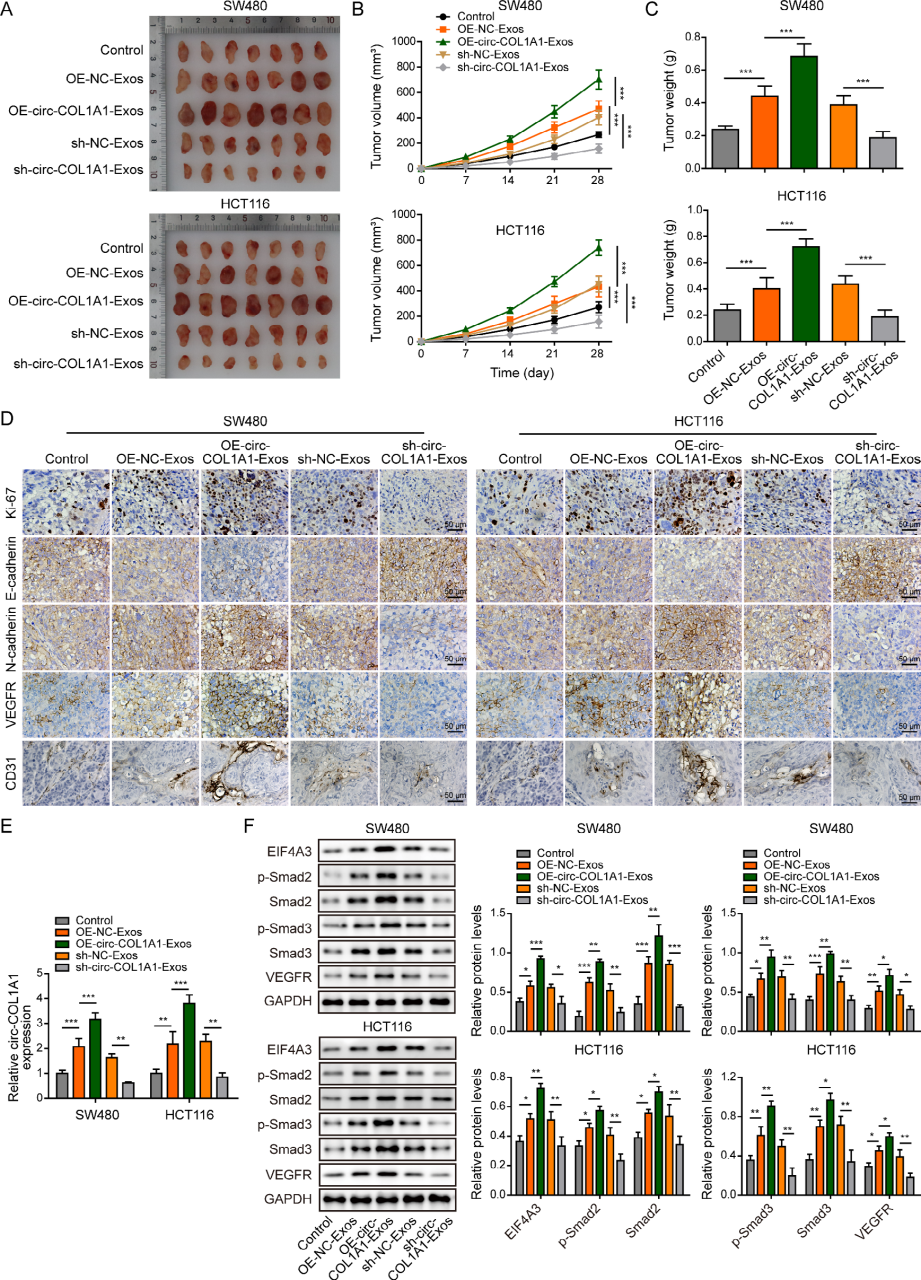
Exosomal circCOL1A1 promotes tumor growth and angiogenesis in vivo. (**A**) Photographs of xenograft tumors. (**B**) Quantitative analysis of tumor volume. (**C**) Quantitative analysis of tumor weight. (**D**) The immunoreactivities of Ki-67, E-cadherin, N-cadherin, VEGFR and CD31were analyzed by IHC. Scale bar, 50 μm. (**E**) The circCOL1A1 levels in xenograft tumors were determined by qRT-PCR. (**F**) The protein levels of EIF4A3, p-Smad2, Smad2, p-Smad3, Smad3, and VEGFR in xenograft tumors were detected by western blot. *, *P* < 0.05, **, *P* < 0.01, ***, *P* < 0.001

We further validated these findings in xenograft mice model. Mice were divided into five groups: control, OE-NC-Exos, OE-circCOL1A1-Exos, sh-NC-Exos and sh-circCOL1A1-Exos. The designated exosomes were administered on mice via tail vein. As presented in Fig. 10A-C, CRC cell-derived OE-circCOL1A1-Exos remarkably accelerated tumor growth in which the tumor volume and weight were much higher than control group. By contrast, sh-circCOL1A1-Exos failed to promote tumor growth. IHC analysis further revealed that the cell proliferation marker Ki-67, the mesenchymal marker N-cadherin, angiogenesis-related molecules VEGFR and CD31 were increased, whereas the epithelial marker E-cadherin was decreased by OE-circCOL1A1-Exos in xenograft tumors. sh-circCOL1A1-Exos exerted opposite effects on these protein levels (Fig. 10D). Moreover, qRT-PCR confirmed that CRC cell-derived exosomes upregulated circCOL1A1 level in xenograft tumors, and OE-circCOL1A1-Exos or sh-circCOL1A1-Exos led to induction or reduction of this molecule in vivo, respectively (Fig. 10E). Similar trends were also observed on the protein levels of EIF4A3, VEGFR, as well as phosphorylated and total Smad2/3 in xenograft tumors (Fig. 10F). These findings suggest that exosomal circCOL1A1 facilitates tumor growth and angiogenesis in vivo.

## Discussion

The incidence of CRC is rising among younger patients worldwide due to westernized diet and sedentary behaviors (Nguyen et al. 2018; Siegel et al. 2021). It is well-accepted that metastatic CRC is associated with two signalings: VEGF and epidermal growth factor receptor (EGFR) pathways (Mousa et al. 2015). Therapeutic agents targeting these pathways have been introduced into routine CRC treatment globally (Mousa et al. 2015). It is of interest to identify biomarkers to predict the patients who will benefit from anti-angiogenic agents. In this study, we demonstrated the pro-angiogenic effects of circCOL1A1 or CRC cell-derived exosomal circCOL1A1 in HUVECs. Mechanistic studies revealed that CRC cell-derived exosomal circCOL1A1 promoted angiogenesis via recruiting EIF4A3 to activate Smad2/3 signaling in vitro and in vivo.

Recently, emerging evidence has illustrated that circRNAs, especially exosomal circRNAs, function as diagnostic and/or prognostic markers in CRC (Li, Wang et al. 2021, Wang et al. 2021). CircRNAs are implicated in cell proliferation, metastasis, angiogenesis, apoptosis and drug resistance (Li, Wang et al. 2021). Our previous findings have demonstrated that circCOL1A1 is elevated in CRC tissues and cells. Silencing of circCOL1A1 suppresses the proliferative and metastatic properties of HCT116 and SW480 cells (Jing et al. 2020). More importantly, GO analysis has further predicted that circCOL1A1 is strongly associated with angiogenesis in CRC (Jing et al. 2020). A recent study has illustrated that circCOL1A1 promotes the phenotype switch of vascular smooth muscle cells (VSMCs) via miR-30a-5p/Smad1/TGF-β axis in atherosclerosis (Ye et al. 2023). In accordance with these reports, our data showed that SW480 or HCT116 cell-derived exosomal circCOL1A1 in particular, promoted angiogenesis of HUVECs. Given the pivotal roles of Smad pathway in angiogenesis, we screened the expression of Smads in HUVECs treated with CRC-derived exosomes. Interestingly, the upregulation of p-Smad2, Smad2, p-Smad3, and Smad3 were observed in HUVECs treated with CRC-derived exosomes. Also, knockdown or overexpression of circCOL1A1 in exosomes reduced or increased the expression levels of phospho- and total Smad2/3, as well as VEGFR, respectively. These data indicate that exosomal circCOL1A1 might facilitate angiogenesis through activating Smad2/3 signaling and upregulating VEGFR in HUVECs. Previous researches have reported that Smad2/3 signaling is indispensable for vascular stability in endothelial cells, and it also contributes to angiogenic resolution by mediating vessel maturation (Itoh et al. 2012, Tian and Schiemann et al. 2009). Our findings firstly reported the critical role of Smad2/3 signaling in exosomal circCOL1A1-enhanced angiogenesis in HUVECs. However, the crosstalk between Smad2/3 signaling activation and VEGFR upregulation needs further investigation.

In addition to the miRNA-sponging activity, circRNAs are implicated in various biological processes via interacting with RNA binding proteins (RBPs) (Du et al. 2017). The RBP EIF4A3 is a component of the exon junction complex, and plays crucial roles in mRNA splicing and metabolism (Lin et al. 2018; Ye et al. 2021). Recent studies have illustrated that EIF4A3 is upregulated in different cancers, including hepatocellular carcinoma (HCC), glioblastoma, pancreatic cancer and CRC (Ye et al. 2021). In CRC, lncRNA H19 recruits EIF4A3 to upregulate the cell cycle-related molecules cyclin D1, cyclin E1 and CDK4, thereby accelerating cell cycle progression and cell growth (Han et al. 2016). Similarly, circ_cse11 decreases PCNA expression by interacting with EIF4A3 in CRC (Xu et al. 2020). In this study, bioinformatics analysis, IHC and qRT-PCR consistently demonstrated that EIF4A3 was upregulated in CRC tissues. RIP, RNA pull-down assay and FISH/IF staining unequivocally showed the direct interaction between circCOL1A1 and EIF4A3 protein. Moreover, functional experiments further revealed that EIF4A3 served as an important effector in exosomal circCOL1A1-enhanced angiogenesis. It is generally accepted that the post-translational modifications play pivotal roles in the expression regulation of proteins (Wang et al. 2023). In the present study, we reported that lack of circCOL1A1 reduced EIF4A3 protein level in HUVECs, we suspected that circCOL1A1 might recruit EIF4A3 protein and regulate its protein expression via post-translational modifications, such as deubiquitination, SUMOylation or glycosylation.

Bioinformatics analysis predicted Smad1/2/3/4/6/7 as the putative binding partners of EIF4A3 protein. Smad2/3 attracted our attention due to their alteration upon circCOL1A1 overexpression or knockdown. Interestingly, Smad2/3 levels were increased in CRC tissues, and CRC patients with high Smad2/3 levels exhibited poor overall survival, indicating the clinical significance of Smad2/3 in CRC. Previous study has illustrated that LINC00667 accelerates NSCLC progression via EIF4A3-stabilized VEGFA (Yang et al. 2020). In pancreatic cancer, LINC01232 modulates TM9SF2 mRNA stability via recruiting EIF4A3 (Li et al. 2019). We thus hypothesized that EIF4A3 might contribute to exosomal circCOL1A1-enhanced angiogenesis via stabilizing Smad2/3 mRNA. As expected, the direct associations between Smad2 mRNA and EIF4A3, as well as between Smad3 mRNA and EIF4A3, were validated by different approaches. Subsequent functional studies confirmed that Smad2/3 pathway played an essential role in exosomal circCOL1A1-enhanced angiogenesis in vitro and in vivo. Interestingly, the migration and tube formation abilities of EIF4A3-knockdown HUVECs were rescued better by Smad2 overexpression. It is possible that Smad2 plays a greater role in EIF4A3-regulated angiogenesis, compared with Smad3. Moreover, it is worth noting that tube formation ability of HUVECs may not only related to the proliferative and migratory capabilities of HUVECs, but also other factors. Therefore, the fully rescued proliferation and migration of HUVECs did not lead to the complete recovery of tube formation ability. Furthermore, we also reported that EIF4A3 bound to Smad2/3 mRNA to maintain their mRNA stabilities, thus inducing Smad2/3 expression. The mechanism by which Smad2/3 pathway regulates angiogenesis in CRC remains elusive. It is well-established that Smad pathway is implicated in the regulation of angiogenesis in many diseases (Chen et al. 2021; He et al. 2023; Hirota et al. 2015; Itoh et al. 2012; Liu et al. 2018). More importantly, Smad signaling also regulates angiogenesis in CRC (Ding et al. 2023; Geng et al. 2013; Itatani et al. 2019; Jin et al. 2017; Li et al. 2022). Previous study has illustrated that Smad2/3 might regulate angiogenesis in CRC through modulating VEGFA expression (Geng et al. 2013), suggesting that circCOL1A1/EIF4A3/Smad2/3 might regulate angiogenesis in CRC through modulating VEGFA expression. Since many research efforts have focused on the Smad2/3-regulated angiogenesis, we therefore focused on the upstream regulatory mechanism of Smad2/3 in this study. The detailed mechanism by which Smad2/3 pathway contributes to angiogenesis merits in-depth investigation in the future study.

In conclusion, we reported that exosomal circCOL1A1 promoted angiogenesis via recruiting EIF4A3 and activating Smad2/3 signaling in CRC. These findings broaden the understanding of angiogenesis in CRC, and exosomal circCOL1A1 might be a promising biomarker for anti-angiogenic therapy.

## Data Availability

All data generated or analysed during this study are included in this published article.

## Abbreviations

CCK-8: Cell Counting Kit-8
circRNA: Circular RNA
CM: Conditioned medium
CRC: Colorectal cancer
EGFR: Epidermal growth factor receptor
EIF4A3: Eukaryotic translation initiation factor 4A3
Exos: Exosomes
FISH: Fluorescence in situ hybridization
GC: Gastric cancer
GO: Gene ontology
HCC: Hepatocellular carcinoma
HUVECs: Human umbilical vein endothelial cells
IF: Immunofluorescence
IHC: Immunohistochemistry
lncRNA: Long non-coding RNA
miRNA: microRNA
NSCLC: Non-small cell lung cancer
NTA: Nanopartical tracking analysis
RBPs: RNA binding proteins
RIP: RNA immunoprecipitation
shRNA: Short hairpin
TCGA: The Cancer Genome Atlas
TEM: Transmission electron microscopy
VEGF: Vascular endothelial growth factor
VSMCs: Vascular smooth muscle cells
